# Microphthalmia-associated transcription factor regulates the visual cycle genes *Rlbp1* and *Rdh5* in the retinal pigment epithelium

**DOI:** 10.1038/srep21208

**Published:** 2016-02-15

**Authors:** Bin Wen, Shuang Li, Huirong Li, Yu Chen, Xiaoyin Ma, Jing Wang, Fan Lu, Jia Qu, Ling Hou

**Affiliations:** 1 Laboratory of Developmental Cell Biology and Disease, School of Ophthalmology and Optometry and Eye Hospital, Wenzhou Medical University, Wenzhou, 325003, China; 2 State Key Laboratory Cultivation Base and Key Laboratory of Vision Science of Ministry of Health and Zhejiang Provincial Key Laboratory of Ophthalmology, Wenzhou Medical University, Wenzhou, 325003, China

## Abstract

Regeneration of the visual pigment by cells of the retinal pigment epithelium (RPE) is fundamental to vision. Here we show that the microphthalmia-associated transcription factor, MITF, which plays a central role in the development and function of RPE cells, regulates the expression of two visual cycle genes, *Rlbp1* which encodes retinaldehyde binding protein-1 (RLBP1), and *Rdh5*, which encodes retinol dehydrogenase-5 (RDH5). First, we found that *Rlbp1* and *Rdh5* are downregulated in optic cups and presumptive RPEs of *Mitf*-deficient mouse embryos. Second, experimental manipulation of MITF levels in human RPE cells in culture leads to corresponding modulations of the endogenous levels of RLBP1 and RDH5. Third, the retinal degeneration associated with the disruption of the visual cycle in *Mitf*-deficient mice can be partially corrected both structurally and functionally by an exogenous supply of 9-cis-retinal. We conclude that the expression of *Rlbp1* and *Rdh5* critically depends on functional *Mitf* in the RPE and suggest that MITF has an important role in controlling retinoid processing in the RPE.

The retinal pigment epithelium (RPE) not only plays critical roles in eye development but also in normal photoreception. It is involved in nutrient and metabolite flow, maintenance of the blood-retinal barrier, ionic homeostasis, regeneration of visual pigment, and phagocytosis of shed photoreceptor outer segments^1^. Not surprisingly, RPE defects lead to photoreceptor dysfunction, retinal degeneration, loss of visual acuity, and blindness^1^,^2^.

Prominent among the many functions of the RPE is its role in the visual cycle, a process responsible for reconstituting 11-cis-retinal from all-trans retinol. After interaction with photons, photoreceptors release all-trans-retinol, which is taken up by RPE cells and converted to an all-trans-retinyl ester by lecithin retinol acyltransferase (LRAT). Subsequently, the isomerase RPE65 converts the all-trans-retinyl ester to 11-*cis* retinol. 11-cis-retinol is then oxidized to 11-cis-retinal by several 11-cis-retinol dehydrogenases, including RDH5 and RDH11. 11-*cis* retinal, which, like 11-cis-retinol, binds RLBP1 (also known as CRALBP), is then released from the RPE and taken up by photoreceptors to reconstitute opsin to the light sensitive rhodopsin^1^.

Mutations in the genes encoding proteins implicated in the visual cycle have recently emerged as the underlying genetic defects responsible for a number of retinal disorders^3^,^4^. Among them, mutations in *Rlbp1* and *Rdh5* are associated with retinal dystrophies and dysfunctions such as retinitis pigmentosa, Newfoundland rod/cone dystrophy, retinitis punctata albescens, and Bothnia dystrophy or fundus albipunctatus^4^,^5^,^6^. To provide a deeper understanding of the role of these two genes, we here focus on the regulation of their expression and identify MITF as one of their major transcriptional regulators.

MITF is a member of the microphthalmia family of transcription factors (MiT). It is expressed at various levels in a variety of cell types, including neural crest-derived melanocytes and RPE cells where it plays prominent roles in development^7^,^8^. In mice, for instance, mutant alleles such as *microphthalmia-vga9* (Mitf mi−vga9) not only cause loss of pigmentation in the skin, but also microphthalmia and retinal degeneration due to abnormalities in RPE structure and function^8^,^9^. Other *Mitf* mutant alleles, such as *microphthalmia-brownish* (*Mitf*^*Mi−b*^) and *microphthalmia-vitiligo* (*Mitf*^*mi−vit*^), however, do not lead to microphthalmia but to postnatal retinal degeneration^10^,^11^. MITF is well known to regulate the expression of tyrosinase and tyrosinase-related protein-1, whose mutations are associated with Oculocutaneous Albinism and Pigmentary Glaucoma^12^,^13^, and of BEST1, whose mutations cause Best Macular Dystrophy^14^. Nevertheless, little is known about MITF’s potential involvement in the regulation of the visual cycle and whether the regulation of visual cycle genes by MITF contributes to retinal degeneration.

Here, we focus on *Rlbp1* and *Rdh5* and their regulation by MITF based on several observations. First, previous results have shown that in *Mitf*-*vit* mutant RPE retinyl ester levels are significantly elevated but that the accumulation of retinyl ester is not due to increased LRAT activity^15^, suggesting that dysregulation of other genes may be involved. Second, preliminary experiments indicated that visual cycle genes are down-regulated in *Mitf*-deficient mouse optic cups and are abundantly expressed in MITF-positive, fully differentiated RPE cells.

Our results show that the visual cycle genes *Rlbp1* and *Rdh5* expression are reduced in the RPE of P11 and optic cups of E12.5 of *Mitf* mutant mouse embryos, and *Rlbp1* and *Rdh5*-expression is missing in *Mitf* mutant embryonic RPE cells. In addition, *RLBP1* and *RDH5* expression directly correlates with experimental manipulation of MITF levels in the human RPE cell line ARPE-19 cells. Finally, addition of exogenous 9-cis-retinal partially rescues retinal degeneration in *Mitf*-deficient mice, suggesting that *Mitf* regulation of visual cycle genes can be functionally relevant for the pathogenesis of retinal degeneration.

## Results

### Impairment of visual cycle gene expression in *Mitf*-deficient retina

**Figure 1:**
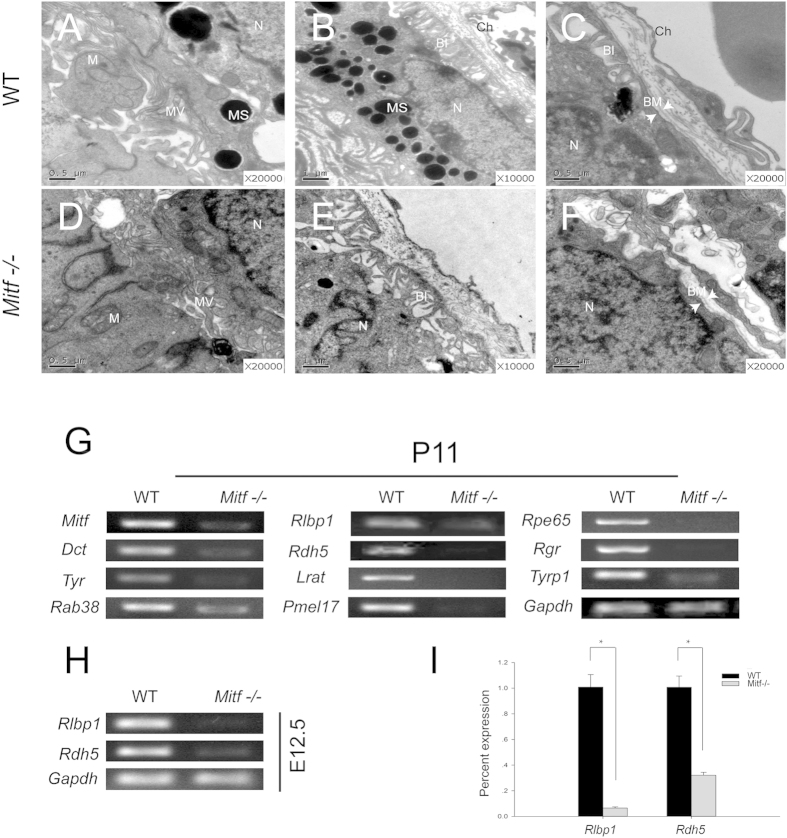
Abnormal RPE ultrastructures and decreased expression of visual cycle genes in *Mitf*−/− mice. (**A–F**) P0 (**A–E**) and P20 (**C,F**) RPE ultrastructures of WT and *Mitf*−/− mice revealed by transmission electron microscopy (TEM). (**A,D**) show the interface between RPE and photoreceptor cells in WT and *Mitf*−/− mice, respectively. The RPE in *Mitf*−/− displayed thickened and shortened microvilli as compared to WT. The microvilli in the apical surface of the RPE are indicated by MV. B and E show the basal part of the RPE in WT and *Mitf*−/−, respectively. Bruch membrane was discontinuous and disintegrated in *Mitf*−/−. (**C,F**) show the basal infoldings of RPE in WT and *Mitf*−/− mice, respectively. The basal infoldings of RPE were sparse and disordered in *Mitf*−/− when compared with that of WT. BI, basal infoldings; BM, Bruch’s membrane; Ch, choroid; M, mitochondria; MS; melanosome; MV, microvilli; N, nucleus. (**G**) RT-PCR analysis of the expression of visual cycle and pigmentation genes in P11 retinas/RPE of WT and *Mitf*−/− mice. (**H**) RT-PCR analysis of the expression of *Rlbp1* and *Rdh5* in E12.5 optic cups of WT and *Mitf*−/− mice. (**I**) Real-time RT-PCR analysis of *Rlbp1* and *Rdh5* expression in WT and *Mitf*−/− optic cups at E12.5. RNA levels of *Rlbp1* and *Rdh5* were normalized to those of *Gapdh*. The experiments were performed in triplicates and the error bars represent the S.E. *indicates P < 0.05. *Gapdh* was used as internal control.

It has been shown previously that RPE development is disrupted in Mitf mi-vga-9 homozygous embryos (hereafter called *Mitf*−/−)^9^,^16^, but the ultrastructure of the mutant RPE has not been examined. Examination by transmission electron microscopy (TEM) revealed that the RPE of *Mitf*−/− P0 and P20 mice displayed apical microvilli and basal infoldings also seen in wild-type RPE but they were sparse and disorganized. In addition, Bruch’s membrane was interrupted and melanosomes and melanin were absent (Fig. 1A–F). These results indicated that although the RPE was retained in *Mitf*−/− mice, its ultrastructure was abnormal.

It has previously been shown that retinyl ester levels were significantly elevated in *Mitf*-*vit* mutant RPE, without, however, showing alterations in LRAT activity^15^. Hence, we first examined the expression of visual cycle genes in wild type and *Mitf*−/− retina/RPE preparations in comparison to the expression of pigmentation-related genes, which are known to be regulated by *Mitf*. At P11, the expression of visual cycle genes *Rlbp1* and *Rdh5* as well as *Lrat, Rpe65,* and *Rgr* were significantly decreased in *Mitf*−/− retina/RPE, as was *Tyr* which encodes the rate-limiting enzyme for melanin synthesis (Fig. 1G). Interestingly, further examination revealed that in wild type, though not in mutant, *Rlbp1* and *Rdh5* were expressed in eye vesicles already at E12.5 when the RPE develops (Fig. 1H,I). In contrast, *Lrat, Rpe65,* and *Rgr* were not expressed at these early stages (not shown). These results suggest that *Mitf* regulates *Rlbp1* and *Rdh5* gene expression both during development and after birth.

### MITF regulates the expression of RLBP1 and RDH5 in RPE cells

**Figure 2:**
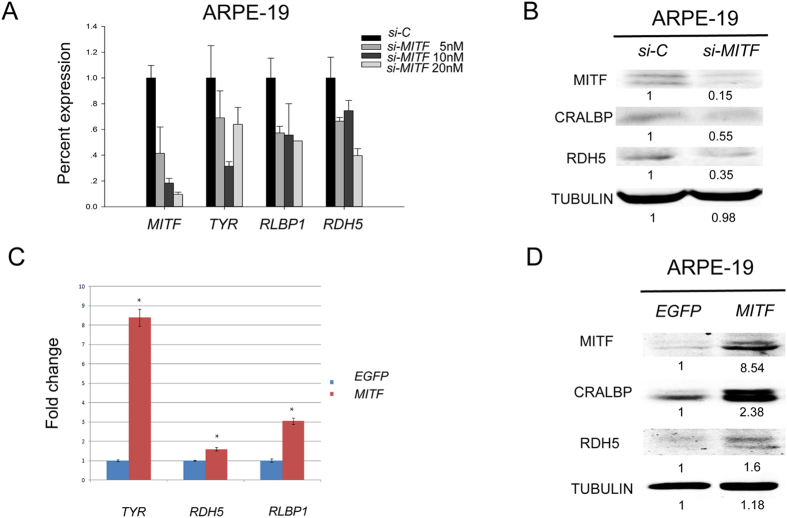
MITF is required and sufficient to induce RLBP1 and RDH5 expression in ARPE-19 cells. (**A**) The expression of *RLBP1* and *RDH5* in ARPE-19 cells transfected with scramble siRNA (si-C) or various concentrations of *MITF* siRNA (si-MITF). Increased si-MITF concentrations resulted in enhanced *MITF* knockdown. The expression of the *MITF* target gene *TYR* was reduced concomitantly with the reduction of *MITF*. With the reduction in *MITF* expression, the expression of *RLBP1* and *RDH5* was also decreased. (**B**) Protein expression of RLBP1 and RDH5 was decreased when MITF was knocked-down in ARPE-19 cells. TUBULIN was used as an internal protein loading control. (**C**) The RNA and protein levels of *RLBP1* and *RDH5* in *EGFP-* and *MITF-*overexpressing ARPE-19. Both RNA and protein expression of RLBP1 and RDH5 were increased when MITF was overexpressed in ARPE19 cells compared to overexpression of *EGFP*. The experiments were performed in triplicate and the error bars represent the S.E. *indicates P < 0.05.

To evaluate whether the regulation of *Rlbp1* and *Rdh5* was indeed controlled by *Mitf*, we tested whether the inhibition or overexpression of MITF can affect the endogenous expression of *RLBP1* and *RDH5* in a human RPE cell line. We first transfected cultures of ARPE-19 cells with si-Mitf, a pool of siRNAs directed against *MITF* or si-NC, a control siRNA. As shown in Fig. 2, knockdown of *MITF* reduced the expression of RLBP1 and RDH5 RNA and protein (Fig. 2A,B). In contrast, overexpression of MITF using a lentiviral expression system increased the expression of RLBP1 and RDH5 proteins (Fig. 2C,D). These results indicate that *MITF* regulates the expression of *RLBP1* and *RDH5* in human RPE cells.

### *Rlbp1* and *Rdh5* expression depends on MITF *in vivo*

**Figure 3:**
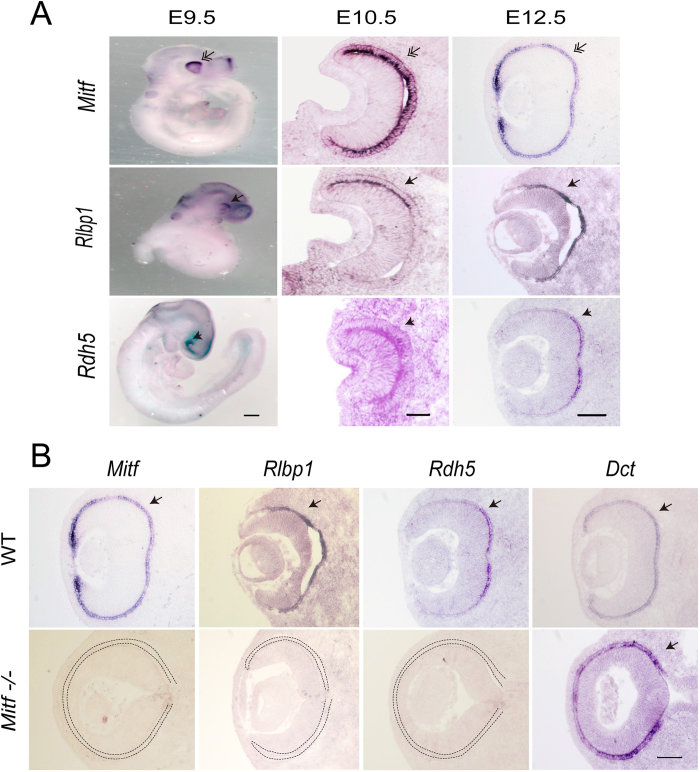
*Rlbp1* and *Rdh5* expression is dependent on *Mitf* during mouse eye development. (**A**) The spatiotemporal expression patterns of *Mitf, Rlbp1* and *Rdh5* during mouse eye development by *in situ* hybridization with an Mitf, Rlbp1, or Rdh5 probe (as indicated on the left-hand side). At E9.5, *Mitf* was expressed in the whole periphery of optic vesicle, with prominent expression in the dorsal part, while *Rlbp1* and *Rdh5* expression were observed exclusively in the dorsal part of optic vesicles (double arrows). *Rdh5* expanded its expression to the presumptive RPE of the optic cup at E10.5, (arrowheads), while *Rlbp1* extended its expression to the presumptive RPE of optic cup at E12.5 (arrows). (**B**) The expression of *Rlbp1* and *Rdh5* in WT and *Mitf*−/− mice embryonic eyes at E12.5 by *in situ* hybridization with an Mitf, Rlbp1, Rdh5, or Dct probe (as indicated on the top). Note that *Rlbp1* and *Rdh5* were expressed in RPE of WT mouse embryos, but their expression was absent in *Mitf*−/−. *Dct* expression, however, remained intact. Scale bar is 50 μm at E10.5, and 100 μm at E12.5.

Although *Rlbp1* and *Rdh5* are expressed in RPE, their spatial and temporal expression patterns during eye development are unclear. By *in situ* hybridization, the expression of *Rlbp1* and *Rdh5* was seen already at E9.5 when the optic vesicle is initially formed (Fig. 3A). At this time point, the expression of these two genes was confined to the dorsal part of the optic vesicle where by comparison *Mitf* expression was highest, but by E12.5 it was extended to the entire presumptive RPE. A comparison between WT and *Mitf*−/− embryos revealed that the expression of *Rlbp1* and *Rdh5* depended on intact *Mitf* while the RPE marker-*Dct* did not (Fig. 3B), confirming previous studies of *Dct* expression^16^. These results suggest that the loss of functional MITF leads to disruption of the expression of visual cycle genes and that *Mitf* is required for the expression of *Rlbp1* and *Rdh5* in mouse RPE.

### 9-cis-retinal treatment partially rescues *Mitf* −/− retinal morphology and function

**Figure 4:**
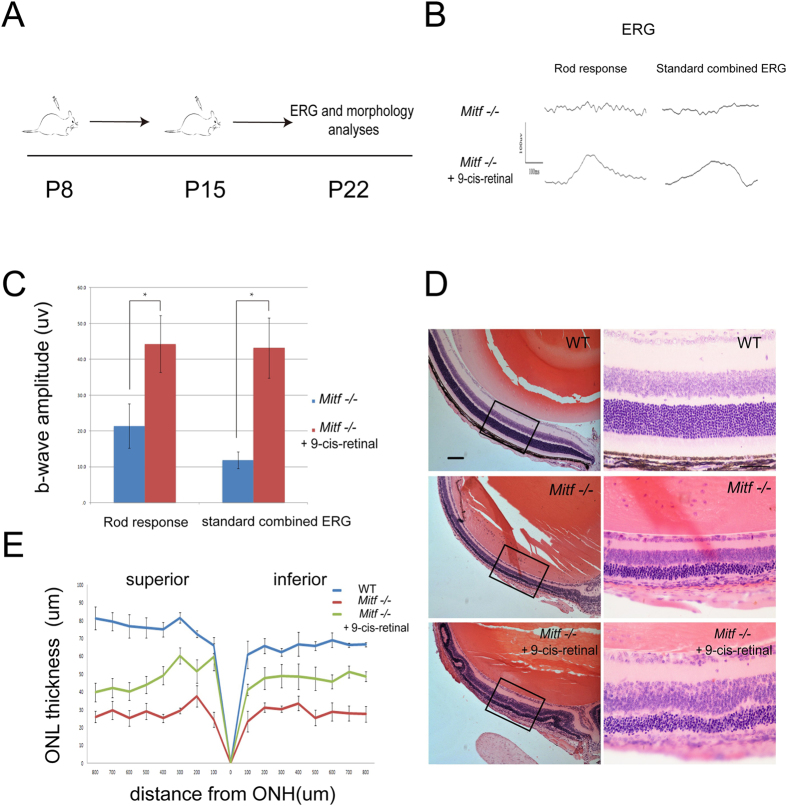
9-cis-retinal treatment partially recovers visual function and retinal structure in *Mitf*−/− mice. (**A**) Schematic representation of 9-cis-retinal administration in mice for this study. Mice were injected subcutaneously with solvent or 9-cis-retinal in the dorsal torso region at both P8 and P15, and were subjected to ERG examination and morphological analysis at P22. (**B**) Representative ERG from *Mitf*−/− mice treated with solvent or 9-cis-retinal at P22. The rod response and standard combined ERG were lost in *Mitf*−/− mice (n = 12) treated with the solvent only. In contrast, both were partially recovered in *Mitf*−/− treated with 9-cis-retinal (n = 12). (**C**) Quantification of b-wave amplitudes from *Mitf*−/− mice treated with solvent (n = 12) or 9-cis-retinal (n = 12) at P22. Note that *Mitf*−/− mice treated with 9-cis retinal showed increased b-wave amplitude in rod responses at −25 dB flash intensity and in standard combined ERG at 0 dB flash intensity. The values are represented by mean and SD and were statistically analyzed by unpaired Student’s t-test. *indicates P < 0.05. (**D**) Histological analysis of WT or *Mitf*−/− eyes after treatment with either solvent or 9-cis-retinal at P22. Compared with WT mice, *Mitf*−/− mice treated with solvent suffered from severe retinal degeneration. The thickness of the outer nuclear layer (ONL) in *Mitf*−/− mice retina was approximately one-fourth of that of wild type, and numbers of nuclei were remarkably reduced. When *Mitf*−/− mice were treated with 9-cis-retinal, however, the retinal thickness was approximately twice that of *Mitf*−/− mice treated with solvent, and the numbers of nuclei were significantly increased compared to those of solvent treated mice. The ONL, inner plexiform layer and ganglion cell layer were partially protected. (**E**) Quantification of the ONL thickness from the optic nerve head (ONH) to the ciliary margin for WT (n = 4) or *Mitf*−/− mice treated with solvent (n = 5) or 9-cis-retinal (n = 7) at P22. Note that the ONL thickness was significantly increased in *Mitf*−/− mice treated with 9-cis-retinal compared with *Mitf*−/− mice treated with solvent.

**Figure 5:**
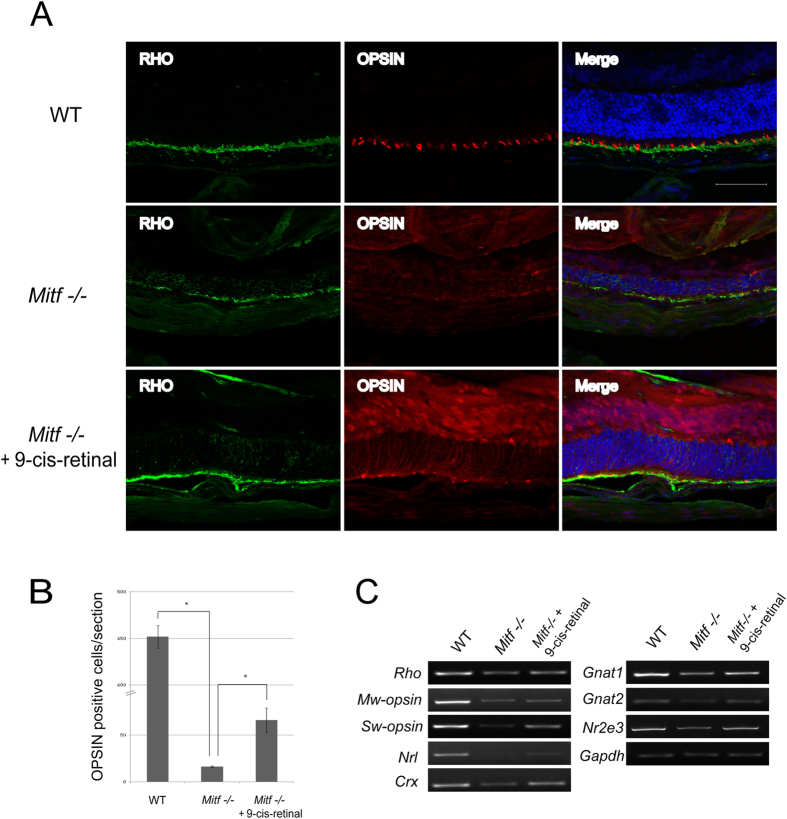
Photoreceptor cells and their related gene expression were maintained in *Mitf*−/− mice treated with 9-cis-retinal. (**A**) RHODOPSIN and OPSIN expression in superior retina of WT and *Mitf*−/− treated with solvent or 9-cis-retinal. Rod RHODOPSIN was localized to the rod outer segment (ROS), and cone OPSIN was localized to the ONL and the rod outer segment in WT neural retina. In *Mitf*−/− mice treated with solvent, the RHODOPSIN and OPSIN localization in ROS were decreased and strikingly mislocalized to the cone cell bodies in the ONL. By contrast, RHODOPSIN and OPSIN localization in ROS, especially Rhodopsin, were maintained in *Mitf*−/− mice treated with 9-cis-retinal. (**B**) Quantification of OPSIN-labeled cells in retinal ROS of WT, *Mitf*−/− treated with solvent or treated with 9-cis-retinal. In contrast to WT, the OPSIN-labeled cells in retinal ROS were almost completely lost in *Mitf*−/− mice treated with solvent, whereas OPSIN-positive cells were partially preserved in *Mitf*−/− mice treated with 9-cis-retinal. (**C**) The expression of photoreceptor marker genes in WT, *Mitf*−/− treated with solvent, and *Mitf*−/− mice treated with 9-cis-retinal at P22 by RT-PCR. For photoreceptor marker genes, 9-cis retinal administration partially restored *S-opsin, M-opsin* and *Gnat2* expression. Moreover, 9-cis retinal administration also restored expression of the rod-specific genes *Rho* and *Gnat1*, as well as genes expressed by both rod and cone cells, including *Crx, Nrl*, and *Nr2e3* expression. Scale bar: 50 μm.

In order to address the question of whether the retinal degeneration in postnatal *Mitf* mutant mice might result in part from impairment of the visual cycle, we made use of the fact that 9-cis-retinal, an analogue of 11-cis-retinal, can rescue retinal degeneration in mice with impaired visual cycle^3^,^17^. Hence, we injected 9-cis-retinal into *Mitf*−/− mice at P8 and P15 and examined their retinal structure and function at P22 (Fig. 4). When injected with solvent alone, the rod response and standard combined electroretinogramm (ERG) was lost in *Mitf* −/− mice (Fig. 4A,B), and the b wave amplitude of the rod response was 21.4 μv and that of the standard combined ERG 11.9 μV (Fig. 4C). Furthermore, the mice showed severe retinal degeneration, marked by loss of most of the outer segments of photoreceptors and loss of the outer and inner plexiform layers (Fig. 4D) and a general reduction in retinal thickness (Fig. 4E). When 9-cis-retinal was injected, however, *Mitf*−/− retinas were markedly improved in ERG and structure, and photoreceptor gene expression (Figs 4 and 5). As shown in Fig. 4C, the average b wave amplitude of rod response was increased to 44.3μv and that of the standard combined ERG to 43.2μV. Compared to untreated mutant, the retinal thickness was double, and the ONL, inner plexiform layer, and ganglion cell layer were partially spared from the degeneration seen in the absence of 9-cis-retinal (Fig. 4D,E).

Previous studies had shown that cone M/L-OPSIN expression was lost in visual cycle gene mutant mice, and that administration of 9-cis-retinal prevented this loss^18^ Therefore, we also examined Opsin and Rhodopsin expression in WT, control *Mitf*−/− and 9-cis-retinal-injected *Mitf*−/− mice. As shown in the corresponding panels in Fig 5A, M/L-OPSIN was normally distributed within the cone outer segments in WT but lost in untreated *Mitf*−/− mice, suggesting cone outer segment degeneration. Administration of 9-cis retinal, however, partially restored M/L-OPSIN expression in the outer segments. As shown in Fig. 5B, the average number of OPSIN positive cells per section was 451 in WT, 16 in untreated *Mitf*−/−, and 66 in treated *Mitf*−/− mice. Furthermore, as shown in Fig. 5C, RT-PCR results indicated that 9-cis retinal administration partially restored the expression of rod genes (*S-opsin, M-opsin*, Rho and *Gnat1/2)* as well as genes expressed by both rods and cones, (*Crx, Nrl*, and *Nr2e)*. Hence, we conclude that 9-cis-retinal can partially bypass the loss of visual cycle gene expression and prevent retinal degeneration in *Mitf* mutant mice.

## Discussion

Here we provide experimental evidence that the expression of two visual cycle genes, *Rlbp1* and *Rdh5*, depends on functional MITF both *in vitro* in a human RPE cell line and *in vivo* in *Mitf* mutant mice. The finding helps explain the previous observation that retinyl ester levels were elevated significantly in the RPE of *Mitf-vit* mutant^15^. In these mutants, the activity of LRAT was not changed so that the increase in retinyl ester was unlikely due to increased production. Rather, it might be due to reduced levels of *Rlbp1* and *Rdh5* as the products of these two genes are involved in processes of the visual cycle downstream of retinyl ester: RLBP1 binds 11-cis-retinol and RDH5 catalyzes the reaction to 11-cis retinal. In *Mitf*−/− embryos, however, *Lrat* expression was reduced, suggesting that retinyl ester levels might not be increased, and hence that the visual cycle was disrupted at even more levels than in *Mitf-vit* mutants.

Our findings clearly show that by regulating visual cycle genes, *Mitf* controls the visual cycle and consequently the reconstitution of functional rhodopsin. They do not, however, answer the question of whether it is the mere interruption of the visual cycle, or a more general damage to the RPE, that leads to the associated retinal degeneration. The RPE, after all, has multiple functions in photoreceptor and retinal physiology through its supply of neurotrophic factors, phagocytosis of photoreceptor outer segments, blood retinal barrier maintenance, etc, and its damage may well interfere with any of these functions.

An experimental approach to distinguish between the role of visual cycle interruption and general RPE damage in the cause of retinal pathophysiology was partially provided by the application of 9-cis-retinal, which previously had been shown to substitute for 11-cis-retinal as the final product of the visual cycle and to rescue retinal degeneration in models of isolated visual cycle interruptions. We found, indeed, that postnatal injection of 9-cis-retinal can partially rescue *Mitf*−/− retinae because gene expression, structure and function were all improved. This was in a way surprising as one might expect that the lack of *Mitf* in the RPE may have so profound developmental effects that the adjacent retina might be permanently damaged. Nevertheless, it is clear that the rescue was only partial. Several explanations may be offered for this observation. First, the supply of 9-cis-retinal may not have reached high enough levels, or may have occurred too late, to fully rescue photoreceptors and retina. Second, as *Mitf* is not only expressed in the future RPE but also in the future retina, albeit at low levels, it was conceivable that the retina might be developmentally impaired even if RPE and the visual cycle were normal. Third, as mentioned above, the restoration of only one function, the visual cycle, might just not be enough to fully normalize the structure and function of a retina that is likely severely damaged at multiple levels by the absence of a normal RPE. Following this latter thought, we would predict that the retinal phenotype associated with certain milder *Mitf* mutations, which might damage the visual cycle but leave other RPE functions largely intact, might more readily be corrected with 9-cis-retinal alone.

In sum, then, our work provides evidence that *MITF* affects the visual cycle at least through the regulation of *RLBP1* and *RDH5*. Recently, it has also been shown that two other transcription factors present in RPE, SOX9 and OTX2, act synergistically to activate the *RLBP1* and *RPE65* promoters^19^. Future work will delineate whether this regulation is direct, operating through enhancers and promoters of the respective genes, or indirect through interspersed factors, what co-operating factors might be involved, and whether the whole set of visual cycle genes is regulated concordantly by *MITF* alone or in conjunction with *SOX9* and *OTX2*.

## Materials and Methods

### Animal

All animal experiments were carried out in accordance with the approved guidelines of the Wenzhou Medical University Institutional Animal Care and Use Committee. C57BL/6J, FVB, and Mitf mi-vga-9 mutant mice were used for this study. To collect mouse embryos at the indicated time points, the noon of the day a vaginal plug was found was defined as embryonic day 0.5 (E0.5).

### Conventional PCR and quantitative real-time PCR

For PCR analysis, mouse retinas were collected at postnatal day 11, and optic cups from mouse embryos were isolated at E12.5. The respective tissues were immediately homogenized in Trizol for RNA extraction. Conventional PCR was first conducted to analyze a set of pigmentation-related genes and visual cycle genes in P11 retina and E12.5 optic cups of wild-type and Mitf mi-vga-9 homozygous mice, and quantitative real-time PCR was performed to confirm expression level alterations of the selected genes. Total RNA was isolated and reverse transcribed into cDNA from mouse retinas, optic cups, or cultured cells as previously described^20^.

### Cell culture, lentivirus preparation and infection

The human retinal pigment epithelial cell line (ARPE-19 cell) was obtained from American Type Cell Culture (ATCC) and maintained in DMEM/F12 medium supplemented with 10% fetal calf serum (Gibco-BRL, Invitrogen). D407 and 293T cells were cultured in DMEM/F12 medium supplemented with 10% fetal calf serum. For lentivirus preparation, 7.5 μg FUW-Mitf, 5.625 μg Pax2 and 1.875 μg PMD plasmids were cotransfected into 293T cells by polyjet transfection reagent according to the instructions of manufacturer (Signagen). After 48h, the supernatant were concentrated by Lenti-X Concentrator (Clontech). Briefly, lentivirus-containing supernatants were mixed with Lenti-X Concentrator at 3:1 ratio, then the mixture was incubated at 4 °C for 30 minutes to overnight. Samples were centrifuged at 1,500 X g for 45 minutes at 4 °C, then off-white pellets were resuspended in DMEM. For lentivirus infection, ARPE19 or D407 cells were seeded in 6-well plate at 1 × 10^5 ^cells per well. After 24 hrs, lentivirus with 8 μg/ml polybrene was added to the culture medium. Six hours after infection, fresh medium was added for 1 hour, and a second round of infection was conducted.

### siRNA transfection

ARPE19 cells were seeded in 12-well plates at 4 × 10^4 ^cells/well before transfection. When the cell density reached 30% confluence, cells were transfected with appropriate dilutions of 20 μM siMitf pool (Thermo scientific) containing 4 siRNAs targeting different regions of MITF RNA, each at 5μM, using lipojet reagent (Signagen) at the indicated concentrations following the company’s instructions. Briefly, for transfection at a final concentration of 20 nM, 20 pmoles siRNA were diluted into 100 μl working solution of LipoJet™ Transfection Buffer, and 1.5 μl LipoJet™ reagent was added and mixed by pipetting up and down. The transfection complex was incubated for 15 min at RT and added to 1 ml medium in each well. Seventy-two hours post-transfection, cells were collected in Trizol or protein lysis buffer. In all experiments, control siRNA (CONTROL Non-Targeting siRNA; Thermo scientific) was included.

### Western blotting

Total protein from Mitf or Control siRNA-treated ARPE19 cells, and EGFP or Mitf-lentivirus-infected ARPE19 cells were subjected to Western blotting with anti-MITF (Abcam, 12039), anti-CRALBP (Thermo scientific, MA1-813), anti-RDH5 (Santa Cruz, sc-98348), anti-TUBULIN (Santa Cruz, sc-5286) antibodies. Western blots were prepared and performed according to a published procedure^20^.

### *In Situ* hybridization

Embryos at various embryonic days as indicated in the results were collected in PBS and fixed overnight at 4 °C using 4% paraformaldehyde (PFA). Sectioning and whole mount *in situ* hybridization were performed according to a published procedure^21^. The *Mitf* and *Dct* riboprobes were described previously^22^,^23^. The *Rlbp1* riboprobe was kindly provided by Dr Connie Cepko (Department of Genetics, Harvard Medical School, Boston, MA). The *Rdh5* riboprobe was obtained by amplifying the 864 bp fragment of the murine *Rdh5* coding region from E12.5 C57BL/6J mouse optic cup cDNA using the sense primers 5- GCC GGC CAG TGA TGC TTT C and the antisense primer 5- CGG GGA AGG ATC CAG GTG AG. The PCR product was cloned into TOPO-TA vector (Invitrogen), and verified by sequencing.

### 9-cis-retinal experiment

For 9-cis-retinal treated experiments, 9-cis-retinal (Sigma) was dissolved in 100% ethanol to generate a 125 mg/ml stock solution. For each experiment, 2 μl of 9-cis-retinal stock was combined with 18 μl ethanol and 180 μl matrigel at 4 °C for a final volume of 200 μl. The mixture was injected subcutaneously into the dorsal torso region as previously described^18^. 9-cis retinal was omitted from the mixture for all control groups. Mitf mi-vga-9 homozygous and heterozygous littermates were injected subcutaneously at P8 and P15, and reared in darkness. Eye balls were collected at P22 for further examinations as described in the results.

### Electroretinography (ERG)

At P22 following solvent or 9-cis-retinal administration, a RETIport system with a custom-built Ganzfeld dome (Roland Consult, Wiesbaden, Germany) was utilized for ERG recording. Overnight dark-adapted mice were anesthetized under dim red light by intraperitoneal injection of 7 μL/g body weight of 6 mg/mL ketamine and 0.9 mg/mL xylazine diluted with 10 mM sodium phosphate, pH 7.2, containing 100 mM NaCl. Pupils were dilated with 1% tropicamide, and a heating pad was used to keep the body temperature at 38 °C. The corneal electrode was a gold wire loop; a reference electrode was placed on the forehead and a ground electrode was applied subcutaneously near the tail. For dark-adapted ERGs, the intensities of flashes were −5.0, −15, −25,and −35 log scotopic candela sec/m2 (cd. s/m2). Twelve ERG scans were averaged for dark-adapted ERGs. Ganzfeld illumination with white light stimulus was applied for a duration of 2 milliseconds. B-wave amplitudes were defined as the difference between the trough and peak of each waveform.

### Immunostaining

For immunostaining, eyes were fixed in 4% PFA at 4 °C for overnight, cryopreserved in 30% sucrose, and embedded in optimal cutting temperature compound. Sections (14 μm) were collected on a cryostat, dried at RT for 30 min, and fixed in 2% PFA for 10 min. After rinsing, the sections were permeabilized with 0.2% Triton-X-100 for 10 minutes and blocked with 10% goat serum for 1 hour at RT. Anti-Rhodopsin (milipore MAB5316) and anti-Opsin (milipore AB5405) were used as primary antibodies, and goat anti-mouse IgG alexa Fluor® 488 (life technologies, A11029) and goat anti-rabbit IgG alexa Fluor® 594 (life technologies, A11037) were used as secondary antibodies. Each staining was performed on slides from at least three animals per condition. Immunostaining results were observed and photographed using a Zeiss confocal microscope.

### Statistical Analysis

Statistical comparisons between two groups were performed by an unpaired Student’s t-test. *P* < 0.05 was considered to be significant. Significant differences between groups are noted by *or **.

## Additional Information

**How to cite this article**: Wen, B. *et al.* Microphthalmia-associated transcription factor regulates the visual cycle genes *Rlbp1* and *Rdh5* in the retinal pigment epithelium. *Sci. Rep.* **6**, 21208; doi: 10.1038/srep21208 (2016).
